# Pathobiology of cancer chemotherapy-induced peripheral neuropathy (CIPN)

**DOI:** 10.3389/fphar.2013.00156

**Published:** 2013-12-18

**Authors:** Yaqin Han, Maree T. Smith

**Affiliations:** ^1^Centre for Integrated Preclinical Drug Development, The University of QueenslandBrisbane, QLD, Australia; ^2^School of Pharmacy, The University of QueenslandBrisbane, QLD, Australia

**Keywords:** chemotherapy-induced peripheral neuropathy (CIPN), mitochondrial dysfunction, oxidative stress, intraepidermal nerve fiber (IENF) degeneration, loss of heat sensitivity

## Abstract

Chemotherapy induced peripheral neuropathy (CIPN) is a type of neuropathic pain that is a major dose-limiting side-effect of potentially curative cancer chemotherapy treatment regimens that develops in a “stocking and glove” distribution. When pain is severe, a change to less effective chemotherapy agents may be required, or patients may choose to discontinue treatment. Medications used to alleviate CIPN often lack efficacy and/or have unacceptable side-effects. Hence the unmet medical need for novel analgesics for relief of this painful condition has driven establishment of rodent models of CIPN. New insights on the pathobiology of CIPN gained using these models are discussed in this review. These include mitochondrial dysfunction and oxidative stress that are implicated as key mechanisms in the development of CIPN. Associated structural changes in peripheral nerves include neuronopathy, axonopathy and/or myelinopathy, especially intra-epidermal nerve fiber (IENF) degeneration. In patients with CIPN, loss of heat sensitivity is a hallmark symptom due to preferential damage to myelinated primary afferent sensory nerve fibers in the presence or absence of demyelination. The pathobiology of CIPN is complex as cancer chemotherapy treatment regimens frequently involve drug combinations. Adding to this complexity, there are also subtle differences in the pathobiological consequences of commonly used cancer chemotherapy drugs, viz platinum compounds, taxanes, vincristine, bortezomib, thalidomide and ixabepilone, on peripheral nerves.

## Introduction

Chemotherapy-induced peripheral neuropathy (CIPN) is a common and potentially dose-limiting side effect of many cancer chemotherapy drug treatment regimens (Burton et al., 2007). The prevalence of CIPN varies from 10 to 100% depending upon the particular anticancer drug or drug combination administered, the dosing regimen, the methods of pain assessment and the particular patient situation (Balayssac et al., 2011). The development of CIPN may result in dose reduction of the cancer chemotherapy agents or a switch to less efficacious agents or even cessation of treatment in the extreme (Gutiérrez-Gutiérrez et al., 2010).

Typically, CIPN presents in patients with a “stocking and glove” distribution in the feet and hands, respectively, due to the vulnerability of the long nerves (Boland et al., 2010). Sensory symptoms that are commonly reported include paresthesia, dysesthesia, allodynia, hyperalgesia, hypoalgesia or pain that is burning, shooting or electric-shock-like (Boland et al., 2010). Painful symptoms may persist well beyond discontinuation of treatment (so called “coasting”) (Quasthoff and Hartung, 2002) resulting in a condition as painful or more painful than the original cancer. Furthermore, although slow recovery of peripheral nerve damage may occur in patients with CIPN, this is not always the case and so pain may persist (Peltier and Russell, 2002).

Anticancer drugs that most commonly induce CIPN are platinum compounds (cisplatin and oxaliplatin), spindle poisons/antitubulins including vincristine and paclitaxel (Wolf et al., 2008; Balayssac et al., 2011), and some newer agents such as the proteasome inhibitor, bortezomib (Hoy, 2013), ixabepilone (Goel et al., 2008) and thalidomide (Kocer et al., 2009). A wide range of solid and hematological malignancies are treated with these compounds and polychemotherapy schedules are used to enhance treatment effectiveness (Cavaletti and Marmiroli, 2010). However, the latter also increase the risk of CIPN (Burton et al., 2007; Argyriou et al., 2013).

The prevalence of cancer is increasing globally with an estimated 17 million new cases projected by 2020 (Kanavos, 2006; Paice, 2011). Cancer survival rates have increased dramatically as new treatments and older therapies are refined to have a greater antitumor effect. This means that the landscape of “cancer pain” has shifted into a form of long term chronic pain in many instances (Burton et al., 2007). In clinical practice, CIPN is poorly diagnosed and under-treated to the detriment of patient quality-of-life and there is no proven method for prevention of CIPN (Balayssac et al., 2011). Although drugs used to provide symptomatic relief of CIPN often lack efficacy and/or have unacceptable side-effects (Balayssac et al., 2005), a recent 5-week randomized, placebo-controlled clinical trial found that oral duloxetine at 60 mg daily produced significant relief of CIPN above placebo (Smith et al., 2013). Despite these promising findings, there is nevertheless a large unmet medical need for novel, well-tolerated analgesic agents to improve relief of CIPN. In the past decade, new insights on the mechanisms underpinning the pathogenesis of CIPN (Balayssac et al., 2011) have been made possible by the advent of rodent models enabling new targets to be identified for use in pain therapeutics discovery programs. Such studies are discussed in the following sections of this review.

## Structural changes in peripheral nerves

**Figure 1:**
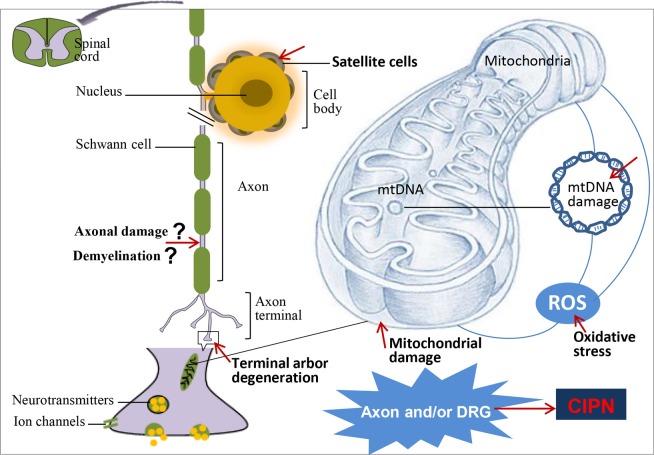
**CIPN pathogenesis and associated morphologic changes**. The neurotoxic effects of cancer chemotherapy agents adversely affect multiple components of the peripheral nervous system (PNS) including axons and cell bodies of dorsal root ganglion (DRG) neurons to cause axonal damage (IENF loss/terminal arbor degeneration), mitochondrial damage and oxidative stress probably associated with inflammation. DRG neurons and their surrounding satellite cells show pathological changes including alterations in levels of expression of multiple ion channels (Xiao et al., 2007; Anand et al., 2010; Kaur et al., 2010; Descoeur et al., 2011), neurotransmitters (Tatsushima et al., 2011), and their receptors (Carozzi et al., 2010b; Mihara et al., 2011), as well as altered gene expression (Alaedini et al., 2008). Mitochondrial dysfunction and IENF loss appear to be important pathobiological features of CIPN that are correlated directly with pain behaviors in rodent models (Flatters and Bennett, 2006; Zheng et al., 2012). Indeed, direct mitochondrial DNA (mtDNA) damage contributes to cisplatin-induced CIPN (Podratz et al., 2011). Myelinated fibers are damaged (Cata et al., 2006) possibly by preferential selection (Dougherty et al., 2004) but the extent to which demyelination is a key pathobiological event is currently unclear.

Cancer chemotherapy agents may differentially affect specific peripheral nervous system (PNS) structures to produce neuronopathy, axonopathy and/or myelinopathy that contribute to the pathogenesis of painful CIPN (Ocean and Vahdat, 2004; Balayssac et al., 2011) (Table 1 and Figure 1).

**Table 1:** **Effects of clinically used cancer chemotherapy agents on peripheral nerve structure in rodent models of CIPN**.

**Chemotherapy agent**	**Dosing regime**	**Rodents**	**PNS tissue examined**	**Extent of peripheral nerve damage**	**References**
Bortezomib	ip, 0.2 mg/kg, 5 consecutive days	Male SD rats	Saphenous nerve	IENF decrease but no degenerating axons	Zheng et al., 2012
			DRGs and IENFs	No DRG neurons with ATF-3 positive nuclei	
	iv, 0.08, 0.15, 0.2, 0.3 mg/kg, 2 or 3 times a week, 4 weeks	Female Wistar rats	Sciatic nerves	Mild to moderate pathological changes involving predominantly Schwann cells and myelin; primarily characterized by myelin sheath degeneration and axonal degeneration. Unmyelinated fibers were unaffected	Cavaletti et al., 2007
	iv, 0.2 mg/kg ×3/week, 4 weeks	Female Wistar rats	Sciatic nerves	No pathological changes in axons and the surrounding myelin sheath	Gilardini et al., 2012
			Optic nerves	Myelin degeneration in a limited number of fibers, optic nerves normal	
	iv, 0.15/0.2 mg/kg × 3/week, 8 weeks	Female Wistar rats	Sciatic nerves	Nerve fiber degeneration, loss of axonal structures in the most severe cases	Meregalli et al., 2010
			DRGs	No morphological alteration in most DRG neurons and satellite cells	
	iv, 0.4/0.8 mg/kg × 2/week, 4 weeks	Female BALB/c mice	DRGs	No pathological changes in DRGs	Carozzi et al., 2010a
			Sciatic nerves	Axonal degeneration in sciatic nerves at higher dose	
	sc, 0.8, 1 mg/kg × 2/week or × 2/week, 6 weeks	Swiss OFI female mice	Sciatic and tibial nerves	Lower density of myelinated large fibers and decreased fiber diameter but no signs of degeneration	Bruna et al., 2010
			Plantar pads		
Cisplatin	ip, 1 mg/kg ×3/week, 2 mg/kg × 2/week, 3 mg/kg ×1/week, 5 weeks	Male SD rats	Lumbar spinal cord Sciatic nerve and paw skin	Myelin sheath remains normal	Authier et al., 2003a
				Unmyelinated fibers were unaffected	
	ip, 3 mg/kg every 3 days, 4 weeks	Male Wistar rats	Sciatic nerves	Degenerated myelinated axons with altered myelin band and altered unmyelinated axons; axonal damage without demyelination	Arrieta et al., 2011
	ip, 2/4 mg/kg × 2/week, 4 weeks	Female BALB/c mice	DRGs	No pathological changes in the DRGs	Carozzi et al., 2010a; Gilardini et al., 2012
		Wistar rats	Sciatic nerves	Mild pathological changes at higher dosage regimen in sciatic nerves	
	ip, 2 mg/kg, 2/week in 4.5 weeks	Male Wistar rats	Sciatic nerves	Focal areas of demyelination and degeneration	Al Moundhri et al., 2013
Oxaliplatin	ip, 2 mg/kg, 5 consecutive days	Male SD rats	Saphenous nerves and IENFs	Oxaliplatin evoked SNCV slowing occurred in the absence of demyelination or degeneration of peripheral nerve axons	Xiao et al., 2012
	ip, 2 mg/kg, 4 alternate days	Male SD rats	Nerve fibers	Significantly fewer IENFs	Boyette-Davis and Dougherty, 2011
	ip, 4 mg/kg, 2/week in 4.5 weeks	Male Wistar rats	Sciatic nerves	Focal areas of demyelination and degeneration	Al Moundhri et al., 2013
	ip, 3, 6 or 12 mg/kg, single	Male SD rats	Lumbar spinal cord	No difference in immunoreactivity for CGRP but substance P was significant higher than for vehicle control group (12 vs. 5%)	Ling et al., 2007
Vincristine	iv, 50, 100 and 150 μg/kg, every second day, up to five injections	Male SD rats	Paw skin	Myelin sheaths remained unaffected	Authier et al., 2003b
	ip, 0.2 mg/kg ×1/week, 5 weeks, 0.1 mg/kg and increase by 0.05 mg/kg each week, 5 weeks	Male rats	Sciatic nerve	Reduction in action potential amplitude associated with axonal degeneration with or without minor changes of segmental demyelination	Ja'afer et al., 2006
Paclitaxel	ip, single 32 mg/kg	Male SD rats	Lumbar spinal cord, Sciatic nerve and paw skin	Axonal degenerative changes while Schwann cells and myelin sheaths remained normal	Authier et al., 2000b
	ip, 0.5, 1, 2, 6 or 8 mg/kg, 4 alternate days	Male SD rats	DRGs	No degeneration, no DRG neurons with ATF-3 positive nuclei	Polomano et al., 2001; Flatters and Bennett, 2006; Bennett et al., 2011
			Sciatic nerves	No degeneration of myelinated or unmyelinated axons	
	iv, 18 mg/kg, D0 and D3	Male SD rats	DRGs	ATF-3 upregulation	Peters et al., 2007
			Sciatic nerve		
	ip, 8 mg/kg × 2/week, 4 weeks	Male Wistar rats	Sciatic nerves	Axonal damage without demyelination	Arrieta et al., 2011
	ip, 16mg/kg × 1/week, 4 weeks iv, 5, 10, 12.5 mg/kg × 1/week, 4 weeks	Female Wistar rats	Axons (sciatic nerve)	Most myelinated fibers have normal histology, some fibers show axonal degeneration	Persohn et al., 2005
	ip, 12.5 mg/kg × 1/week, 9 weeks	Female Wistar rats	DRGs	Increased immunohistochemical staining for ATF-3	Jamieson et al., 2007
	iv, 10 mg/kg × 1/week, 4 weeks	Female Wistar rats	Sciatic nerves	No pathological changes in axons and surrounding myelin sheath	Gilardini et al., 2012
			Optic nerves		
	iv, 18 mg/kg, twice, every 3 days	Male SD rats	Trigeminal ganglia	Increased immunohistochemical staining for ATF-3	Jimenez-Andrade et al., 2006
			DRGs		
	ip, 4.5 mg/kg, 25 mg/kg, or 60 mg/kg	Female C57BL/6 mice	Sciatic nerves	Macrophage-mediated demyelination, axons completely stripped of their myelin sheaths and surrounded by the cytoplasm of debris-filled phagocytes in some cases	Mo et al., 2012
	ip, 8 or 16 mg/kg × 1/week, 5 weeks	Female Wistar rats	Sciatic/peroneal nerves and DRGs	Decrease in number of large myelinated fibers, but not due to a reduction in myelin thickness, mild axonal loss with minimal demyelination	Cavaletti et al., 1995
	iv, 50.70 mg/kg, × 1/week, 4 weeks	Female BALB/c mice	DRGs	No pathological changes	Carozzi et al., 2010a
			Sciatic nerves		
	ip, 30 mg/kg once or several times at different intervals	BDF1 mice	Dorsal funiculus	Nerve fiber degeneration characterized by axonal and myelin fragmentations and phagocytosis	Mimura et al., 2000
			Dorsal spinal roots		
			Peripheral nerves		

ATF, activating transcription factor; CGRP, calcitonin gene-related peptide; DRG, dorsal root ganglia; IENFs, intraepidermal nerve fibers; iv, intravenous injection; ip, intraperitoneal injection; sc, subcutaneous; SD, Sprague-Dawley; SNCV, sensory nerve conduction velocity.

Cancer chemotherapy-induced peripheral nerve injury appears to be due primarily to axonopathy (McDonald et al., 2005; Persohn et al., 2005; Gilardini et al., 2012) that is seen both in patients with CIPN (Cata et al., 2007; Burakgazi et al., 2011) and in rodent models of CIPN (Cavaletti et al., 2007; Boyette-Davis et al., 2011). Thus, peripheral nerve degeneration or small fiber neuropathy is generally accepted as underpinning the development of CIPN (Ling et al., 2010; Boyette-Davis et al., 2011; Burakgazi et al., 2011; Wang et al., 2012).

### The longest axons are the first affected

Peripheral nerves contain a variety of nerve fibers that differ in their respective morphology, degree of myelination, function and biochemical features (Gutiérrez-Gutiérrez et al., 2010). These various fiber types are differentially sensitive to the neurotoxic effects of cancer chemotherapy agents with the longest nerves having the greatest vulnerability (Wilkes, 2007; Gutiérrez-Gutiérrez et al., 2010). This may be related to their higher metabolic requirements (Chen and Chan, 2006; Mironov, 2007). Clinically, symptoms develop initially in the feet and hands, followed by proximal progression to the ankles and wrists in a “stocking and glove” distribution (Lomonaco et al., 1992; Wolf et al., 2008).

### Mylelinated fibers are damaged with/without altered myelin structure whereas unmyelinated fibers are mostly unaffected

Myelin is a lipid- and protein-rich sheath that insulates axons and facilitates faster conduction of nerve impulses compared with unmyelinated axons (Gilardini et al., 2012). Although myelinated fibers are damaged (Cata et al., 2006), perhaps even by preferential selection (Cavaletti et al., 1995; Dougherty et al., 2004), the extent to which demyelination is a key pathobiological event in CIPN is unclear. For example, using X-ray diffraction capable of detecting even subtle changes in the myelin structure, there were no structural alterations in the myelin sheath of the sciatic and optic nerves in rat models of CIPN induced using cisplatin, paclitaxel or bortezomib (Gilardini et al., 2012). These findings mirror the findings of earlier work that used fixed tissues (spinal cord and DRGs) from rodents administered the same cancer chemotherapy agents (Cavaletti et al., 1995) as well as from humans with paclitaxel-induced CIPN (Postma et al., 1995). In patients with bortezomib-induced CIPN, approximately 50% had pure small fiber neuropathy whereas the remainder had mixed small and large fiber involvement (Richardson et al., 2009).

In rat models of paclitaxel, cisplatin and bortezomib-induced CIPN, there were no clear-cut changes in the structure of internodal myelin (Gilardini et al., 2012). However, higher dosages of bortezomib were associated with an increased risk of peripheral nerve degeneration and possibly demyelination in contrast to lower dosages that nevertheless induced neuropathic pain behaviors (Zheng et al., 2012) (Table 1). In earlier work in patients administered paclitaxel, sural nerve biopsy revealed severe nerve fiber loss, axonal atrophy (with absence of axonal regeneration) and secondary demyelination (Sahenk et al., 1994). These peripheral nerve changes argue more for ganglionopathy than axonopathy as the most likely structural change in paclitaxel-induced neurotoxicity (Sahenk et al., 1994).

### Slowing of SNCV may not be due to demyelination or degeneration of peripheral nerve axons

In CIPN, reduced sensory nerve conduction velocity (SNCV) (Gilardini et al., 2012; Xiao et al., 2012), can only be attributed reliably to myelinopathy if it is associated with preserved nerve compound action potentials (Gilardini et al., 2012). Unfortunately, the technical limitations of current neurophysiological methods do not allow the relative contributions of demyelination and axonal degeneration on reduced SNCV in CIPN to be assessed (Gilardini et al., 2012). In rats with docetaxel-induced CIPN, reduced levels of myelin and mRNA encoding myelin suggest that myelin is targeted in experimental peripheral neuropathies (Roglio et al., 2009). These findings are consistent with observations of taxane-induced axonal damage and secondary demyelination (Sahenk et al., 1994; Quasthoff and Hartung, 2002; Windebank and Grisold, 2008). The extent to which individual anticancer agents or treatment combinations induce differential structural changes in peripheral nerves, is currently unclear. This is a knowledge gap that requires systematic investigation in rodent models for comparison with the changes observed in skin biopsy specimens from patients with CIPN.

### IENF loss without degeneration of peripheral nerve axons and associated with mitochondrial dysfunction

Unmyelinated fibers and terminal nerve arbors are major sites of cancer chemotherapy-induced neurotoxicity (Grisold et al., 2012) such that intraepidermal nerve fiber (IENF) loss or terminal arbor degeneration is proposed as a common lesion in various toxic neuropathies (Bennett et al., 2011; Zheng et al., 2012).

In a rodent model of paclitaxel-induced CIPN, significant IENF degeneration was not apparent by approximately 10 days after initiation of the paclitaxel treatment regimen (2 mg/kg on 4 alternate days) with peak effects observed several days later (Xiao et al., 2011). IENF degeneration and the development of pain behavior appear to be linked as both have similar delays to onset and peak effects (Xiao et al., 2011). Using electron microscopy at the time of peak pain severity, there were no signs of axonal degeneration in the saphenous nerve of these animals at a level just below the knee joint (Flatters and Bennett, 2006). Additionally, upregulation of activating transcription factor-3 (ATF-3) expression, a marker of axonal injury (Tsujino et al., 2000), was not observed in the nuclei of afferent neurons (Flatters and Bennett, 2006). Similar findings have been observed in rat models of vincristine, oxaliplatin and bortezomib-induced CIPN such that neuropathic pain behaviors were associated with IENF degeneration in the absence of peripheral nerve axonal degeneration (Aley et al., 1996; Tanner et al., 1998; Topp et al., 2000; Siau and Bennett, 2006; Bennett et al., 2011).

Clinically, there is IENF loss in patients with CIPN (Boyette-Davis et al., 2011; Giannoccaro et al., 2011) despite these individuals having normal peripheral nerve axon counts (Holland et al., 1998; Herrmann et al., 1999) and normal nerve conduction results (Periquet et al., 1999; Devigili et al., 2008; Løseth et al., 2008). This led Holland et al. (1998) to coin the term “terminal axonopathy” that is akin to the more recently promulgated “terminal arbor degeneration” concept (Bennett et al., 2011). In patients, an increase in the swelling ratio of IENFs appeared to be predictive of a decrease in IENF density and this was correlated with the severity of painful neuropathy induced in the feet by paclitaxel (CIPN), diabetes, AIDS, and idiopathic neuropathy (Schmidt et al., 1997; Lauria et al., 2003). However, administration of much larger doses of cancer chemotherapy agents in rats, such as paclitaxel either as a single bolus (12.5–32 mg/kg) (Authier et al., 2000b; Jamieson et al., 2007) or as cumulative doses (8 and 16 mg/kg once-weekly for 5 weeks) (Cavaletti et al., 1995) or bortezomib at 2.4–4.8 mg/kg (Cavaletti et al., 2007; Meregalli et al., 2010; Gilardini et al., 2012), resulted in degeneration of peripheral nerve axons and DRG neurons, together with ATF-3 up-regulation in DRG neurons (Jamieson et al., 2007; Peters et al., 2007). Thus, the extent to which peripheral nerve axons are damaged by chemotherapy agents appear to be directly related to the dosing regimen (Table 1).

Comparatively high concentrations of paclitaxel are found in the DRGs relative to peripheral nerve and spinal cord (Herrmann et al., 1999), that may be underpinned by the fact that the subepidermal axon bundles in peripheral nerves lack a perineurium (a component of the blood-nerve barrier). Additionally, anterograde transport of paclitaxel from sensory neuron cell bodies to the IENFs would take time for toxic levels to be reached in the terminal arbors (Bennett et al., 2011). Such a lag period may potentially explain the coasting effect, i.e., the delay between treatment cessation relative to the loss of IENFs and the appearance of pain hypersensitivity (Bennett et al., 2011).

IENF degeneration and abnormal spontaneous discharge of primary afferent nerve fibers in rat models of CIPN may be underpinned by mitochondrial dysfunction and consequent energy deficiency (Boyette-Davis and Dougherty, 2011; Xiao et al., 2012; Zheng et al., 2012). Mitochondria are concentrated in regions of high metabolic demand (Chen and Chan, 2006; Mironov, 2007) such as sensory terminal boutons that are packed with mitochondria (Breathnach, 1977; Ribeiro-Da-Silva et al., 1991; Bennett et al., 2011). The high energy requirement of the intraepidermal terminal arbor is thought to be due, at least in part, to the constant degeneration and regeneration (re-modeling) of the arbor in its ever changing microenvironment (Bennett et al., 2011). This is because the epidermis is in a continuous state of renewal with a total epidermal turnover time of approximately 45 days in humans (Bergstresser and Taylor, 1977).

## Mitochondrial dysfunction and oxidative stress

Mitochondria are the energy-generating structures in cells with their dysfunction implicated in the pathogenesis of cancer and a range of neurodegenerative diseases (Florea and Büsselberg, 2011). Abnormalities in mitochondrial structure and function in peripheral sensory nerve fibers are postulated as key CIPN mechanisms and appear to be correlated directly with pain behavior (Flatters and Bennett, 2006; Zheng et al., 2012). In multiple myeloma patients administered cycles of bortezomib in combination with dexamethasone, bortezomib toxicity on mitochondria resulted in impairment of the electrogenic Na^+^-K^+^-ATPase-dependent pump resulting in axonal membrane depolarization that preceded axonal degeneration (Nasu et al., 2013). In patients with vincristine and bortezomib-induced CIPN, there were significant changes in the expression of genes involved in the control of mitochondrial function in myeloma plasma cells and peripheral blood (Broyl et al., 2010). Interestingly, exposure of cultured DRG neurons to cisplatin and paclitaxel *in vitro* induced mitochondrial damage that was reversed by pretreatment with the antioxidant, α-lipoic acid (Melli et al., 2008). Additionally, the development of CIPN in rodent models (Table 2) and patients (Table 3) can be prevented by treatment with drugs that enhance mitochondrial function. Conversely, as mitochondrial poisons exacerbate neuropathic pain behaviors in rodent models of CIPN (Xiao and Bennett, 2012), CIPN appears to be linked to mitotoxicity (Figure 1).

**Table 2:** **Summary of pharmacological agents that enhance mitochondrial function as well as prevent and/or alleviate CIPN in rodent models**.

**Pharmacological agent**	**Rodent model**	**Efficacy outcome**	**Dose and route**	**References**
Acetyl-L-carnitine (antioxidant)	Paclitaxel	+ (intervention)	100 mg/kg, p.o. Daily ×10	Flatters et al., 2006
	Paclitaxel	+(prophylactic)	50 and 100 mg/kg, p.o. Daily ×21	Flatters et al., 2006
	Paclitaxel	+ (prophylactic and intervention)	100 mg/kg, s.c. Daily	Ghirardi et al., 2005
	Vincristine	+ (prophylactic and intervention)	100 mg/kg, s.c. Daily	Ghirardi et al., 2005
	Cisplatin	+ (prophylactic and intervention)	100 mg/kg, s.c. Daily	Ghirardi et al., 2005
	Oxaliplatin	+ (prophylactic and intervention)	100 mg/kg, s.c. Daily	Orlando et al., 2005
	Oxaliplatin	+ (prophylactic)	100 mg/ml/kg, p.o. Daily	Xiao et al., 2012
Olesoxime	Paclitaxel	+ (prophylactic)	3 or 30 mg/kg, p.o. Daily	Xiao et al., 2009
	Oxaliplatin	+ (prophylactic)	30 mg/ml/kg, p.o. Daily	Xiao et al., 2012
Silibinin (antioxidant)	Oxaliplatin	+ (prophylactic)	100 mg/kg, p.o. Daily	Di Cesare Mannelli et al., 2012
Allopregnanolone	Oxaliplatin	+ (prophylactic and intervention)	2 or 4 mg/kg, Every 2 or 4 days	Meyer et al., 2011

p.o., per os; s.c., subcutaneous.

**Table 3:** **Clinical trial evidence for the role antioxidants in the relief of CIPN**.

**Medications**	**Patients involved**	**Chemotherapy agent**	**Trial**	**Efficacy**	**Weather interfere with anticancer efficacy**	**References**
α-Lipoic acid (Treatment)	14	Docetaxel and isplatin	Randomised	Yes	–	Gedlicka et al., 2003
	15	Oxaliplatin	–	Yes	–	Gedlicka et al., 2002
Acetyl-L-carnitine (Treatment)	25	Cisplatin and/or Paclitaxel	–	Yes	–	Bianchi et al., 2005
	27	Cisplatin and/or Paclitaxel	–	Yes	–	Maestri et al., 2005
	409	Taxane-based	RCT	No; pain worsened	–	Hershman et al., 2013
Glutathione (Prevention)	31	Cisplatin	Randomized	Yes	No	Colombo et al., 1995
	151	Cisplatin	–	Yes	–	Smyth et al., 1997
	27	Oxaliplatin/5-fluorouracil/leucovorin (FOLFOX)	Randomized	Yes	No	Milla et al., 2009
	52	Oxaliplatin-based	RCT	Yes	–	Cascinu et al., 2002
Amifostine (Prevention)	92	Oxaliplatin (FOLFOX4)	Randomized	Yes	No	Lu et al., 2008
	187	Paclitaxel and Carboplatin	Randomized	yes	–	Lorusso et al., 2003
	27	Cisplatin and Paclitaxel	–	Not really	–	Moore et al., 2003
	38	Paclitaxel and Carboplatin	Randomized	Yes	–	Kanat et al., 2003
	72	Paclitaxel and Carboplatin-based	RCT	Yes	±	Hilpert et al., 2005
Org 2766 (Prevention)	196	Cisplatin and cyclophosphamide	–	No	–	Roberts et al., 1997
	55	Cisplatin and cyclophosphamide	RCT	Yes	No	van et al., 1990
N-acetylcysteine (Prevention)	14	Cisplatin-based	Randomized placebo controlled	Yes	–	Lin et al., 2006

RCT, Randomized, Double-Blind, Placebo-Controlled Trial.

### Mitotoxicity

#### Direct mitochondrial DNA (mtDNA) damage

Cisplatin forms adducts with mitochondrial DNA resulting in direct mitochondrial DNA (mtDNA) damage that is a novel mechanism for cisplatin-induced CIPN and is distinct from the established nuclear DNA (nDNA) damage pathway (Podratz et al., 2011). DRG neurons accumulate high levels of cisplatin-DNA adducts both *in vitro* and *in vivo* (McDonald et al., 2005; Ta et al., 2006) such that the cisplatin concentration in the PNS is comparable with that in tumor tissue (Gregg et al., 1992; Screnci and McKeage, 1999; Melli et al., 2008).

Cisplatin-DNA adducts can be removed and DNA repaired by the nucleotide excision repair (NER) system that is present in nDNA (McDonald et al., 2005; Podratz et al., 2011), in contrast to mtDNA where the NER system is absent (Croteau et al., 1999). Hence, cisplatin-mtDNA adducts inhibit mtDNA replication and mtRNA transcription to cause mitochondrial degradation (Podratz et al., 2011) in DRG neurons.

#### Increased mitochondrial swelling and vacuolation in peripheral nerve axons

In rat models of paclitaxel, oxaliplatin and bortezomib-induced CIPN, the number of swollen and vacuolated mitochondria in the axons of A- and C-primary afferent sensory nerve fibers was significantly higher (37.3 and 152%, respectively) than for vehicle-treated control rats (Xiao et al., 2011, 2012; Zheng et al., 2012). These changes resulted in mitochondrial dysfunction characterized by significant deficits in mitochondrial respiration and ATP production that were rescued by prophylactic treatment with acetyl-L-carnitine. The latter is an acetylated derivative of the natural amino acid, L-carnitine, that has an essential role in the transport of long-chain free fatty acids into mitochondria (Zheng et al., 2011, 2012). Interestingly, there was a relative sparing of mitochondria in the corresponding peripheral nerve Schwann cells (Flatters and Bennett, 2006; Zheng et al., 2011, 2012; Xiao and Bennett, 2012; Xiao et al., 2012).

In DRG satellite cells, bortezomib induced intracytoplasmic vacuolation characterized by damage to mitochondria and the endoplasmic reticulum (Cavaletti et al., 2007). These changes appear to be underpinned by activation of the mitochondrial-based apoptotic pathway including caspase activation (Broyl et al., 2010; Lee et al., 2012) as well as dysregulation of calcium homeostasis (Landowski et al., 2005). Paclitaxel-induced mitochondrial damage was confined to the axons of primary afferent sensory with sparing of motor neurons (Xiao et al., 2011). The high and persistent exposure of primary sensory neuron cell bodies in the DRGs to paclitaxel may contribute to this selective effect (Xiao et al., 2011).

#### Opening of the mPTP and dysregulation of calcium homoeostasis

Paclitaxel opens the mitochondrial permeability transition pore (mPTP), a multi-molecular complex containing a voltage-dependent anion channel that induces mitochondrial calcium release (Kidd et al., 2002; Flatters and Bennett, 2006). Acetyl-L-carnitine can prevent mPTP opening (Pastorino et al., 1993) and is associated with a reduction in paclitaxel, oxaliplatin and bortezomib-induced CIPN when administered prophylactically in rodents (Jin et al., 2008; Bujalska and Makulska-Nowak, 2009; Carozzi et al., 2010b; Xiao et al., 2012; Zheng et al., 2012).

Mitochondria have a large calcium buffering capacity and so impaired calcium uptake or increased calcium leakage from mitochondrial stores may have a pathological role in CIPN (Jaggi and Singh, 2012). This notion is supported by the fact that vincristine-induced neurotoxicity in rats was reversed by drugs that reduce elevated intra-neuronal calcium concentrations (Muthuraman et al., 2008; Kaur et al., 2010). In other work, increased expression levels of the α_2_δ subunit of voltage-gated Ca^2+^ channels in the DRGs were correlated with the development of mechanical allodynia (Luo et al., 2001). Conversely, drugs that bind to the α_2_δ subunit such as gabapentin (Flatters and Bennett, 2004; Xiao et al., 2007) and pregabalin (Saif et al., 2010; Nakashima et al., 2012; Peng et al., 2012), as well as the L-type calcium channel blocker, lercanidipine (Saha et al., 2012), showed efficacy for prevention of CIPN in rodent models and patients (Nguyen and Lawrence, 2004; Saif et al., 2010; Nakashima et al., 2012).

A retrospective review of 69 patients administered oxaliplatin concluded that calcium channel blockers reduce CIPN (Tatsushima et al., 2013). Although intravenous Ca^2+^/Mg^2+^ infusions reportedly attenuate the development of oxaliplatin-induced CIPN without compromising cancer treatment efficacy (Wolf et al., 2008; Kurniali et al., 2010; Wen et al., 2013), there are lingering concerns regarding a negative effect on cancer chemotherapy treatment efficacy. Hence, this needs to be evaluated for each class of cancer chemotherapy agent (Kurniali et al., 2010).

### Oxidative stress

In a rat model of oxaliplatin-induced neuropathy, markers of oxidative stress including lipid peroxidation, carbonylated proteins, and DNA oxidation increased in the systemic circulation, the sciatic nerve and the lumbar spinal cord (Di Cesare Mannelli et al., 2012), with these changes prevented by antioxidant treatment (Di Cesare Mannelli et al., 2012; Nasu et al., 2013). Similarly, production of reactive oxygen species (ROS) was increased by cisplatin (Florea and Büsselberg, 2011), and bortezomib (Wang et al., 2011). In patients receiving docetaxel for the treatment of cancer, the occurrence of grade ≥2 CIPN was more frequent in individuals homozygous for *GSTP1* ^105^Ile allele, that encodes glutathione S-transferase pi 1 (GSTP1), an enzyme involved in the regulation of oxidative stress (Mir et al., 2009).

A role for oxidative stress in the pathobiology of CIPN is supported by multiple *in vitro* and *in vivo* studies showing that antioxidants have neuroprotective effects in CIPN (Table 2). In particular, the non-specific ROS scavenger, phenyl N-tert-butylnitrone (PBN), administered according to an intervention protocol in rats administered paclitaxel, attenuated development of mechanical (Kim et al., 2010) and cold hypersensitivity in the hindpaws (Fidanboylu et al., 2011). Conversely, for rats administered auranofin, a compound that increased oxidative stress, oxaliplatin and paclitaxel-induced neuropathic pain behaviors were exacerbated (Xiao and Bennett, 2012). Furthermore, as the superoxide-specific scavenger, TEMPOL (4-hydroxy-2,2,6,6-tetramethylpiperidine-1-oxyl) neither alleviated established paclitaxel-induced CIPN nor prevented its development in rodents, ROS but not superoxide radicals alone, are implicated in CIPN pathogenesis (Fidanboylu et al., 2011).

Although a benefit of antioxidants for the treatment and/or prevention of CIPN has been shown in multiple clinical studies (Table 3), most did not report on their impact on anticancer efficacy, and so this is a knowledge gap.

Increased spinal dorsal horn levels of peroxynitrite in rats with paclitaxel-induced CIPN (Doyle et al., 2012) implicate a role for reactive nitrogen species (RNS) in CIPN pathogenesis (Kamei et al., 2005; Mihara et al., 2011). Augmented peroxynitrite production may occur via two mechanisms with the first involving activation of nitric oxide synthase and NADPH oxidase to induce formation of the peroxynitrite precursors, NO and SO (Doyle et al., 2012). The second involves inactivation of the enzyme (manganese superoxide dismutase) that catalyzes peroxynitrite degradation (Doyle et al., 2012). This latter mechanism is supported by observations that peroxynitrite decomposition catalysts (FeTMPyP^5+^ and MnTE-2-PyP^5+^) prevented development of neuropathic pain behaviors in rat models of paclitaxel, oxaliplatin and bortezomib-induced CIPN (Doyle et al., 2012; Janes et al., 2013).

CIPN-induced nitro-oxidative stress results in increased production of proinflammatory cytokines (TNF-α and IL-1β), reduced production of anti-inflammatory cytokines (IL-10 and IL-4), as well as post-translational nitration of glutamate transporters and glutamine synthetase in astrocytes, the net result of which is enhanced pro-nociceptive glutamatergic signaling (Doyle et al., 2012). Treatment strategies that shift the balance in favor of anti-inflammatory cytokines have potential for slowing the development and progression of peripheral neuropathy in patients receiving cancer chemotherapy drugs (Wang et al., 2012).

## Loss of heat sensitivity in CIPN

### Diverse results of heat sensitivity in CIPN

Primary afferent nerve fibers affected by cancer chemotherapy drug treatment regimens often exhibit both positive and negative sensory phenomena resulting in altered nociceptive thresholds (Nahman-Averbuch et al., 2011). Increased nociceptive thresholds may develop due to nerve fiber loss whereas reduced nociceptive thresholds may develop as a result of peripheral and central sensitization (Nahman-Averbuch et al., 2011).

In general, there is heat hypoalgesia or a loss of heat sensitivity in patients with CIPN (Dougherty et al., 2004; Cata et al., 2006; Attal et al., 2009; Nahman-Averbuch et al., 2011) as well as in most rodent models of this condition (Authier et al., 2000a,2003a; Fischer et al., 2001; Cata et al., 2006, 2008; Garcia et al., 2008; Hori et al., 2010; Xiao et al., 2012; Zheng et al., 2012). Additionally, cold allodynia is a characteristic symptom of painful CIPN in patients (Cata et al., 2006) as well as in rodent models (Authier et al., 2003a,b; Cata et al., 2006; Xiao et al., 2012).

### Loss of heat sensitivity may result from sensitization/desensitization of TRPV1

Loss of heat sensibility may be due to myelinated A-fiber damage and loss of transient receptor potential vanilloid 1 (TRPV1)-expression (Woodbury et al., 2004) C-fibers (Dougherty et al., 2004).

A small increase in ROS production activates transcriptional machinery to enhance TRPV1 expression levels in C-fibers (Suzukawa et al., 2000; Kishi et al., 2002; Schmeichel et al., 2003). Additionally, nerve growth factor (NGF) facilitates increased TRPV1 expression by nociceptive C-fibers and directly increases the number of neurons that respond to noxious heat (Stucky and Lewin, 1999; Amaya et al., 2004). Enhanced thermal sensitivity results from sensitization (phosphorylation) of TRPV1, transduced by protein kinase C (PKC) (Kamei et al., 2001; Di Marzo et al., 2002; Hong and Wiley, 2005) and/or mitogen-activated protein kinases (MAPK) (Ji et al., 2002; Clapham, 2003). In the DRGs and hindpaw skin of hyperalgesic and hypoalgesic mice, TRPV1 expression levels are increased and decreased, respectively (Pabbidi et al., 2008). Thermal hypoalgesia may be underpinned by reduced TRPV1 expression and function, that in turn may lead to more serious complications (Pabbidi et al., 2008).

Other TRP channels implicated in the pathogenesis of CIPN include TRPA1 that is expressed by nociceptors and is activated by oxidative stress. The transient benefit of the TRPA1 antagonist HC-030031 in mice with bortezomib or oxaliplatin-induced CIPN, suggests a role for early activation/sensitization of TRPA1 by oxidative stress by-products in establishment of CIPN (Trevisan et al., 2013). Additionally, TRPV4 may contribute to paclitaxel-induced mechanical hypersensitivity in CIPN (Alessandri-Haber et al., 2004), whereas TRPA1 and TRPM8 over-expression were induced in the DRGs by oxaliplatin (Anand et al., 2010; Descoeur et al., 2011). Cisplatin and oxaliplatin-induced neurotoxicity of DRG neurons in rats results in p38 MAPK and ERK1/2 activation as well as a reduction in JNK/Sapk phosphorylation (Scuteri et al., 2009, 2010). Apart from the foregoing, a broad array of other molecular mechanisms have been implicated in the pathobiology of CIPN and these have been reviewed elsewhere (Jaggi and Singh, 2012; Wang et al., 2012) and are summarized in Table 4.

**Table 4:** **Molecular mechanisms implicated in the pathogenesis of CIPN**.

**Chemotherapy agents**	**Rodent CIPN models and human studies**	**Mechanism**	**References**
Cisplatin	Male C57BL6 mice	Up-regulation of TRPV1, TRPA1 and TRPM8	Anand et al., 2010; Ta et al., 2010;
Oxaliplatin	Female Wistar rats-cultured DRGs	TRPM8 and/or TRPA1 over-expression; respond to cold allodynia	Descoeur et al., 2011; Goswami, 2012
Cisplatin	Male SD rats	Activation of p38 MAPK and ERK1/2, along with downregulation of SAPK/JNK in cultured DRGs	Scuteri et al., 2010
Oxaliplatin			
Vincristine	Male SD rats	Calcium increase either by influx of extracellular Ca^2+^ or release from mitochondrial intracellular stores, binding to α_2_δ subunit of Ca^2+^ channel; decreased calcium flux	Xiao et al., 2007; Kaur et al., 2010
Paclitaxel			
Paclitaxel	Human neuroblastoma cell line, SHSY-5Y	Activation of calpain, degradation of neuronal calcium sensor (NCS-1), and loss of intracellular calcium signaling	Benbow et al., 2012
Paclitaxel	Female/male Wistar rats	NMDA receptor antagonists antagonize CIPN in prevention but not intervention protocol or only at high doses	Pascual et al., 2010; Mihara et al., 2011
Vincristine	Male SD rats		
Cisplatin			
Oxaliplatin			
Bortezomib			
Oxaliplatin	Male mice- C57BL6J	DNA damage	Brederson et al., 2012; Ta et al., 2013
Cisplatin	Male SD rats		
Vincristine			
Oxaliplatin	Male SD rats	Increase in PKC activity in supra-spinal regions	Norcini et al., 2009
Paclitaxel but Not Oxaliplatin	Male SD rats- cultured DRG	Increased release of substance P and altered CGRP and somatostatin release	Tatsushima et al., 2011
Cisplatin	Female patients	Decrease in NGF levels by Total Neuropathy	Cavaletti et al., 2002, 2004
Paclitaxel	Female Wistar rats	Score (TNS) in patient and in rat plasma samples	
Oxaliplatin	Patients	Dysfunction of axonal Na^+^ channels	Park et al., 2011;
	Rats	Dysfunction of axonal K^+^ channels	Kagiava et al., 2013
Vincristine	Female Inbred C57BL mice	Increase in 5-HT_2A_ receptors in dorsal horn and DRGs	Hansen et al., 2011
Paclitaxel	Male C57BL/6 mice	Antagonists of Kinin B1 and B2 receptors attenuate CIPN	Costa et al., 2011
Cisplatin	Male SD rats	Activation of cannabinoid CB2 receptors	Deng et al., 2012
Paclitaxel			
Paclitaxel	Female WT and ϱ_1_-KO CD-1mice	Antagonists of the sigma-1 receptor attenuate CIPN	Nieto et al., 2012
Oxaliplatin	Patients	Integrin beta-3 L33P is related to CIPN severity but not the development of CIPN	Antonacopoulou et al., 2010
Paclitaxel	Male SD rats	Inflammation	Alaedini et al., 2008; Wang et al., 2012
Cisplatin			
Taxol	Balb/c mice	Increased glial fibrillary acidic protein expression in satellite glial cells, and gap junction-mediated coupling between satellite glial cells	Warwick and Hanani, 2013
Oxaliplatin			
Oxaliplatin	Male SD rats	Activation of spinal astrocytes accompanied by increased expression of astrocyte-astrocyte gap junction connections via Cx43	Yoon et al., 2013
		Activation of drug transporters (nervous system transporters including glutamate, copper transporters, etc.)	Ceresa and Cavaletti, 2011
		Patient's genetic background	Windebank and Grisold, 2008; Broyl et al., 2010; Grisold et al., 2012

CGRP, Calcitonin gene related peptide; IENFs, intraepidermal nerve fibers; MAPK, mitogen activated protein kinase; NMDA (N-methyl-D-aspartate) receptors; TRPV, transient receptor potential vanilloid.

## Between cancer chemotherapy agent differences in the pathobiology of CIPN

CIPN affects sensory nerves predominantly; while motor, autonomic or CNS (Schlegel, 2011) involvement is rare (Grisold et al., 2012). Sensory nerves allow the perception of touch, pain, temperature (small fiber); position, and vibration (large fiber) (Wilkes, 2007). The persistent cumulative injury caused by cancer chemotherapy agents most often affects sensory nerve cell bodies in the DRGs (e.g., cisplatin) and/or the afferent and efferent axons lying outside the spinal cord (e.g., paclitaxel, oxaliplatin) (Quasthoff and Hartung, 2002).

It is generally assumed that platinum compounds irreversibly bind to DNA thereby inducing apoptosis of primary sensory neurons (Velasco and Bruna, 2010). Antitubulins (paclitaxel, docetaxel and vincristine) bind to microtubules, interrupt axonal transport, target the soma of sensory neurons as well as nerve axons, to induce neuronal death (Bennett, 2010; Cavaletti and Marmiroli, 2010; Velasco and Bruna, 2010). In cultured rat DRG neurons, paclitaxel increased the release of the pro-nociceptive neuropeptide, substance P, whereas oxaliplatin did not; the extent to which this difference contributes to differences in paclitaxel and oxaliplatin-induced peripheral nerve neurotoxicity, remains to be determined (Tatsushima et al., 2011). In patients with CIPN, sensory testing shows that peripheral nerve abnormalities appear to have distinct features depending upon the cancer chemotherapeutic agent involved (Cata et al., 2006), but the mechanistic basis remains unclear (Gilchrist, 2012).

Conversely, it is also likely that one or more pathobiologic mechanisms are shared among anticancer agents (Dougherty et al., 2004; Grisold et al., 2012; Zheng et al., 2012). For example, nerve biopsies from rodents and patients administered cisplatin (Dougherty et al., 2004), paclitaxel, oxaliplatin, vincristine, and bortezomib show similar morphological changes (loss of IENFs) even though these compounds have different neurotoxic targets (Flatters and Bennett, 2006; Bennett et al., 2011; Boyette-Davis et al., 2011; Burakgazi et al., 2011; Pachman et al., 2011; Xiao et al., 2012; Zheng et al., 2012). Additionally, mitotoxicity appears to be a factor in common in the pathobiology of CIPN induced by the taxane, paclitaxel, the platinum-complex agent, oxaliplatin, and the proteasome-inhibitor, bortezomib, in rodent models (Zheng et al., 2011, 2012; Xiao et al., 2012).

Although CIPN may share mediators in common with other types of neuropathic pain, the disparity in efficacy of anti-neuropathic agents suggests underlying mechanistic differences (Farquhar-Smith, 2011). For example, NGF deficiency in peripheral nerves is a phenomenon in common between cisplatin-induced CIPN (Cavaletti et al., 2002) and early diabetic neuropathy (Anand, 2004). Hypersensitivity to heat is common in the CCI-rat model of neuropathic pain, but it is very minor or absent in rat models of CIPN (Bennett, 2010) and in patients with either CIPN (Dougherty et al., 2004; Hershman et al., 2011) or diabetic neuropathy (Sorensen et al., 2006; Nahman-Averbuch et al., 2011). Such dissociations indicate that the pathophysiological mechanisms responsible for peripheral nerve injury and neuropathic pain are at least in part dependent upon the cause of the nerve injury (Bennett, 2010).

## Conclusion

CIPN is characterized by multiple sensory changes including the development of (i) mechanical allodynia, whereby light pressure or touch that would normally be perceived as innocuous, evokes pain, (ii) cold allodynia whereby cold temperature evokes a painful sensation, (iii) slowing of SNCV, and (iv) loss of heat sensitivity.

Although the precise pathobiology of CIPN remains to be fully elucidated, recent research implicates “terminal arbor degeneration” (Bennett et al., 2011) and the associated mitochondrial dysfunction and mitotoxicity (Podratz et al., 2011; Zheng et al., 2012) as well as oxidative stress (Nasu et al., 2013). Additional investigation is required to better define subtle between-chemotherapy agent differences in the pathogenesis of CIPN as a means for enhancing rational discovery of novel treatments with potential to prevent and/or attenuate the development of CIPN.

### Conflict of interest statement

The authors declare that the research was conducted in the absence of any commercial or financial relationships that could be construed as a potential conflict of interest.
